# Toward precision acupuncture: phlegm-dampness pattern as a candidate metabolic–microbial endotype in posterior circulation ischemic vertigo: a narrative review

**DOI:** 10.3389/fneur.2026.1876704

**Published:** 2026-08-24

**Authors:** Yike Han, Ruili Wang, Yichen Ma, Lijin Chen, Ting Zhang

**Affiliations:** 1 School of Acupuncture-Moxibustion and Orthopedics, Hubei University of Chinese Medicine, Wuhan, Hubei, China; 2 Acupuncture and Moxibustion Center, Hubei Provincial Hospital of Traditional Chinese Medicine, Wuhan, Hubei, China; 3 Hubei Provincial Clinical Research Center for Acupuncture and Moxibustion in Obesity Treatment, Wuhan, Hubei, China; 4 Acupuncture and Moxibustion Center, Affiliated Hospital of Hubei University of Chinese Medicine, Wuhan, Hubei, China; 5 Hubei Shizhen Laboratory, Wuhan, Hubei, China; 6 Hubei Key Laboratory of Theory and Application Research of Liver and Kidney in Traditional Chinese Medicine, Wuhan, Hubei, China

**Keywords:** acupuncture, endotype, gut–brain axis, neuroinflammation, phlegm-dampness pattern, posterior circulation ischemia, precision medicine

## Abstract

Posterior circulation ischemic vertigo (PCIV) denotes vertigo attributable to ischemia in the vertebrobasilar territory. It overlaps with the vascular vertigo categories of the Bárány Society diagnostic criteria and may indicate posterior circulation stroke or transient ischemic attack, so timely vascular evaluation is essential. In traditional Chinese medicine (TCM) theory, phlegm-dampness is regarded as a principal pathological product in vertigo, and pattern-based prescribing frequently targets it; whether phlegm-dampness pattern in PCIV corresponds to a biologically defined patient subgroup is unknown. This narrative review critically appraises the evidence behind that possibility. We searched PubMed, Embase, Web of Science, the Cochrane Library, China National Knowledge Infrastructure (CNKI), and Wanfang from inception to June 30, 2026. Direct clinical evidence in PCIV is limited to trials and meta-analyses of low certainty indicating that acupuncture, alone or as an adjunct, increases vertebrobasilar blood-flow velocity and improves symptom scores; transcranial Doppler velocity is not equivalent to tissue perfusion, and the composite “clinical effective rate” is a non-standardized outcome. Biomarker studies link phlegm-dampness constitution—assessed in non-PCIV cohorts—to dyslipidemia, low-grade inflammation, and gut microbiome alterations including depletion of *Flavonifractor plautii* and its product phytosphingosine. Mechanistic studies, conducted almost entirely in healthy volunteers or animal models, suggest that acupuncture can modulate cerebrovascular hemodynamics, inflammatory and oxidative-stress signaling, and gut–brain communication. Integrating these strands, we propose a candidate metabolic–microbial endotype framework as a hypothesis-generating model rather than an established entity: the proposed abnormalities have not been shown to coexist in pattern-defined PCIV patients, nor to predict differential response to acupuncture. Pattern-stratified trials with pre-specified biomarker panels are needed to test the framework.

## 1 Introduction

Ischemic events in the vertebrobasilar territory account for approximately 20–25% of all ischemic strokes and disproportionately present with vertigo, dizziness, and gait instability rather than the focal motor deficits that dominate anterior-circulation syndromes (1–3). Posterior circulation ischemic vertigo (PCIV) is widely used in the literature—particularly in the Chinese clinical literature—to describe such vertigo-dominant presentations of vertebrobasilar ischemia, yet the term lacks a uniform operational definition; Section 3 defines how it is used in this review and relates it to contemporary diagnostic criteria for vascular vertigo. Vertigo-predominant posterior circulation events are frequently missed at initial emergency evaluation, and non-contrast CT is insensitive to acute posterior fossa ischemia within the first 24 h (4, 5). Conventional pharmacological management with antiplatelet agents, vasodilators, and vestibular suppressants reduces acute symptoms but offers incomplete protection against recurrence and is constrained by adverse effects (1).

Traditional Chinese medicine (TCM) has long managed vertigo through pattern differentiation. Within classical theory, phlegm-dampness is considered a principal pathological product in vertigo, summarized in the historical aphorism “no phlegm, no vertigo” and in the Danxi school’s framing of phlegm as central to vertigo pathogenesis (6–8). This theoretical tradition explains why phlegm-dampness is frequently targeted in pattern-based prescribing for vertigo, including PCIV; it does not, by itself, constitute clinical evidence about the frequency or biology of any pattern in PCIV, a distinction we maintain throughout this review.

A separate line of modern research has examined the biological correlates of phlegm-dampness constitution—a relatively stable predisposition category in TCM constitution theory—using metabolic, immune, transcriptomic, and microbiome profiling (9–11). In parallel, mechanistic studies, mostly in healthy volunteers and animal models, indicate that acupuncture can engage cerebrovascular hemodynamics, NF-κB and NLRP3 inflammasome signaling, Nrf2-mediated antioxidant defense, and gut–brain communication (12–15). Whether these constitution-associated biomarker findings apply to PCIV patients carrying a phlegm-dampness disease pattern, and whether acupuncture’s mechanisms differentially benefit such patients, has not been directly studied.

**Figure 1:**
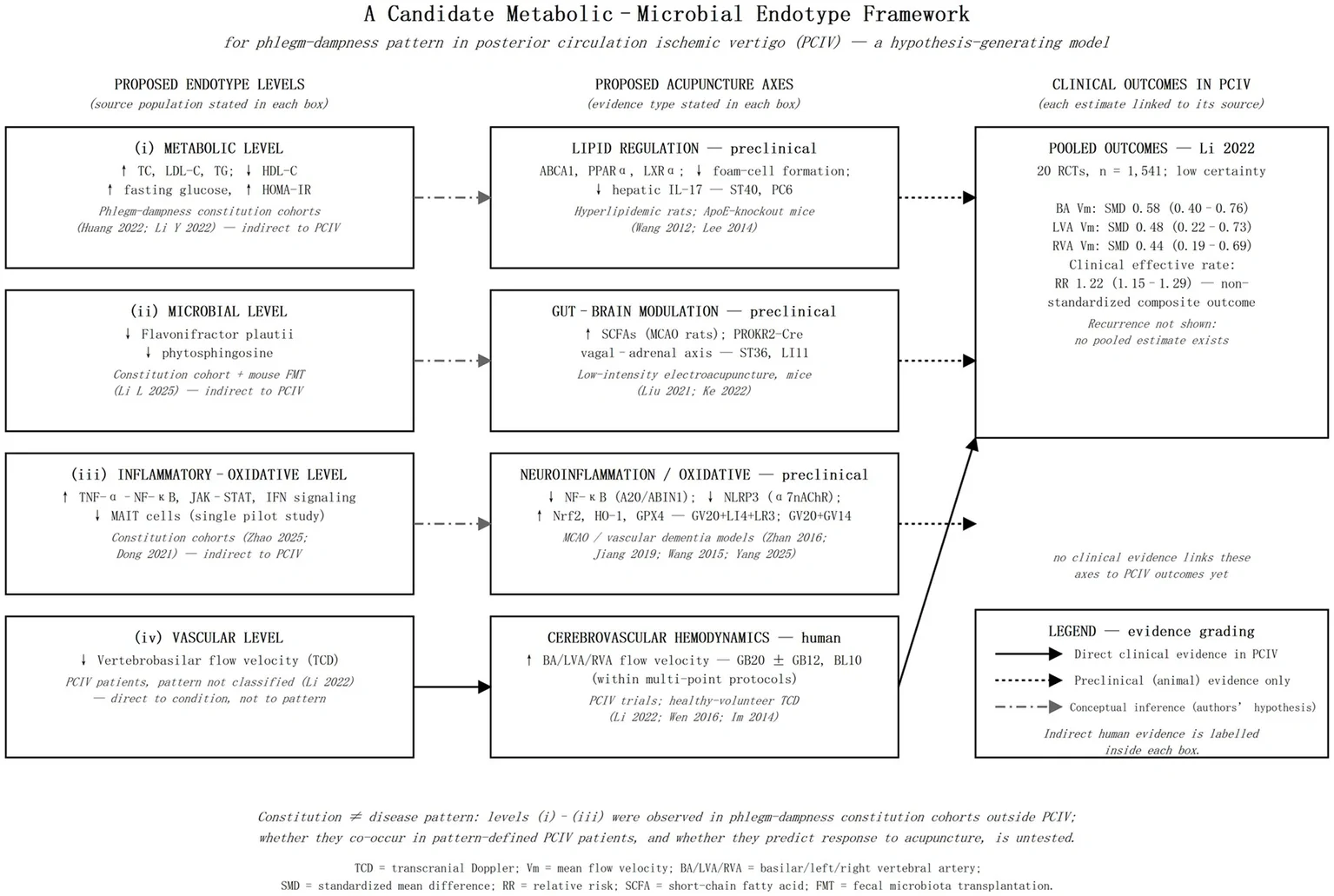
A candidate metabolic–microbial endotype framework for phlegm-dampness pattern in posterior circulation ischemic vertigo (PCIV), presented as a hypothesis-generating model. Left column: The four proposed endotype levels, derived predominantly from phlegm-dampness constitution cohorts outside PCIV (levels i–iii) and from PCIV patients without pattern classification (level iv). Centre column: The proposed acupuncture mechanistic axes, labelled by evidence type. Right column: Clinical outcomes reported in PCIV, with each estimate linked to its source; all pooled estimates are from Li 2022 (20 RCTs, *n* = 1,541): basilar artery *SMD* = 0.58 (95% CI 0.40–0.76); left vertebral artery *SMD* = 0.48 (0.22–0.73); right vertebral artery *SMD* = 0.44 (0.19–0.69); clinical effective rate *RR* = 1.22 (1.15–1.29), a non-standardized composite outcome. Recurrence is not shown because no pooled recurrence estimate exists. Line types grade the evidence behind each link: solid = direct clinical evidence in PCIV; dotted = preclinical (animal) evidence only; grey dash–dot = conceptual inference (authors’ hypothesis); indirect human evidence (constitution cohorts, healthy volunteers) is labelled inside each box. BA, basilar artery; LVA, left vertebral artery; RVA, right vertebral artery; SMD, standardized mean difference; RR, relative risk; CI, confidence interval; SCFA, short-chain fatty acid; PPARα, peroxisome proliferator-activated receptor α; MAIT, mucosal-associated invariant T cell; ABIN1, A20-binding inhibitor of NF-κB activation 1; α7nAChR, α7 nicotinic acetylcholine receptor; HO-1, heme oxygenase-1; NQO1, NAD(P)H quinone oxidoreductase 1; GPX4, glutathione peroxidase 4; TCD, transcranial Doppler; NO, nitric oxide.

The present review therefore critically appraises, rather than assumes, the proposition that phlegm-dampness pattern in PCIV might correspond to a candidate metabolic–microbial endotype—a mechanistically defined subgroup nested within the clinical phenotype (Figure 1). Three questions structure the review. First, what is PCIV, and what clinical evidence bears on the distribution of TCM patterns in this condition? Second, what is the biological substrate of phlegm-dampness constitution, and how directly does it apply to PCIV? Third, what is the clinical evidence for acupuncture in PCIV, through which mechanisms might it act, and how should a candidate endotype framework be tested in future biomarker-guided trials? Throughout, we label each body of evidence by its source population and its directness to PCIV, and we present the endotype framework explicitly as a hypothesis to be tested, not an established conclusion.

## 2 Methods

The preparation and self-assessment of this narrative review were guided by the Scale for the Assessment of Narrative Review Articles (SANRA) (16). We searched PubMed, Embase, Web of Science, the Cochrane Library, CNKI, and Wanfang from inception to October 31, 2025, with a formal update search of all six databases completed on June 30, 2026, covering every topic of the review. The search combined three Boolean concept blocks: (i) acupuncture-related terms (acupuncture, electroacupuncture, moxibustion, manual acupuncture, acupoint; 针灸, 电针, 针刺); (ii) condition-related terms (posterior circulation ischemia, vertebrobasilar insufficiency, ischemic vertigo, cervical vertigo, dizziness, posterior circulation infarction; 后循环缺血, 椎基底动脉供血不足, 眩晕, 颈性眩晕); and (iii) mechanism- and pattern-related terms (phlegm-dampness, Tanshi, gut microbiota, short-chain fatty acid, NF-κB, NLRP3, Nrf2, cerebral blood flow, vagal, endotype, metabolic syndrome; 痰湿, 证型, 体质, 肠道菌群). English and Chinese terms were used in the English- and Chinese-language databases respectively, and no language restrictions were applied. Reference lists of included articles and relevant systematic reviews were screened for additional sources. The constitution-based biomarker studies (Section 5) and the non-PCIV mechanistic studies (Section 7) were identified through supplementary targeted searches of the same databases using construct- and pathway-specific terms, together with the reference-list screening described above, rather than through the combined three-block strategy alone. A representative complete search strategy (PubMed) is provided in Supplementary Table S1.

Eligibility principles were as follows. For clinical evidence, we included randomized controlled trials, systematic reviews, and meta-analyses of acupuncture in PCIV; because PCIV-specific evidence is sparse, studies in cervical or cervicogenic vertigo were included only as explicitly labelled indirect evidence (Section 6.2) and were not pooled or interpreted together with PCIV evidence. For biomarker evidence, we included human studies of phlegm-dampness constitution or pattern irrespective of disease status, and we report the source population of each study so that indirectness to PCIV is visible (Table 1). For mechanistic evidence, given the very large acupuncture literature, we prioritized primary studies that directly examined acupuncture’s effect on pathways nominated by the human biomarker findings (hemodynamics, NF-κB/NLRP3, Nrf2, gut–brain signaling), together with systematic reviews of preclinical work; where such studies were conducted in healthy volunteers or animal models, this is stated where the evidence is presented. We therefore prioritized peer-reviewed primary studies, systematic reviews and meta-analyses, and mechanistic preclinical work. When two sources addressed the same question, we preferred more recent and primary sources. Each included study was appraised individually for design, reporting quality, relevance, and limitations; indexing status of the publishing journal was not used as a selection criterion. Where evidence depended on individual trials, we reported effect sizes and confidence intervals as published rather than reproducing point estimates of secondary endpoints. Titles and abstracts, and subsequently full texts, were screened independently by the first two authors; disagreements were resolved by discussion, with the corresponding author arbitrating when consensus was not reached. All references were verified against the version of record (authors, title, journal, year, volume, pages or article number, and DOI), all numerical results quoted in the text, tables, and figure were checked directly against the original publications, and the correspondence between in-text citations and the reference list was checked in full.

**Table 1:** Biomarker studies of phlegm-dampness constitution or pattern: populations, instruments, key findings, and directness to PCIV. None of the studies enrolled PCIV patients.

Study	Population (disease status)	Constitution or pattern; instrument	*n*	Key findings	Major limitations/confounders	Directness to PCIV
Huang et al. (11)	Cardiology outpatients and health-screening attendees, Sichuan (mixed disease status)	Constitution; Wang Qi constitution criteria (physician-scored)	74 PDC vs. 66 balanced	↑ GLU, TC, TG, LDL-C; ↓ HDL-C; combined GLU + TG + LDL-C + lymphocyte model AUC 0.878	AUC is in-sample (no split, CV, or external validation); PDC group had more diabetes (41.9% vs. 10.6%) and CAD (15.6% vs. 1.5%)	Indirect (constitution, not PCIV)
Li et al. (30)	Community adults with PDC, Beijing; stratified by metabolic state	Constitution; ZYYXH/T157-2009	32 (11 normal indices; 8 prediabetes; 13 marginal lipids)	DIA-PRM proteomics: lipid/inflammation pathway shifts; B2MG validated across groups; subclinical dyslipidemia in index-normal subjects	Pre–post herbal intervention without placebo control; very small subgroups	Indirect (constitution, not PCIV)
Zhao et al. (10)	Adult volunteers (constitution/subhealth cohort), Shanghai	Constitution; four examinations + Tanshi questionnaire	scRNA-seq 4 PDC vs. 9 non-PDC; FACS 5 vs. 5	↓ MAIT cells (FACS-confirmed); ↑ CD14 + monocytes; heightened TNF-α–NF-κB, JAK–STAT, interferon signaling; ↑ hypoxia/apoptosis programs	Pilot scale; Treg finding failed FACS replication; sex imbalance; cross-sectional	Indirect (constitution, not PCIV)
Dong et al. (31)	Disease-free health-examination attendees, Beijing	Constitution; CCMQ (ZYYXH/T157-2009)	Discovery 13 PDC vs. 9 balanced; validation 9 vs. 12	398 differentially expressed lncRNAs, 437 mRNAs enriched in lipid-metabolism and immune–inflammation pathways; 5 lncRNA–mRNA pairs confirmed by RT-qPCR	Cross-sectional association only; small samples; no mechanistic experiments	Indirect (constitution, not PCIV)
Li et al. (9)	Community cohort, Beijing (92.2% of PDC with ≥1 metabolic disorder)	Constitution; ZYYXH/T157-2009	167 PDC vs. 42 balanced (metagenomic subset 40 vs. 10)	↓ *Flavonifractor plautii* and ↓ serum phytosphingosine (correlated with PDC score; random-forest CV-AUC 0.85); FMT from PDC donors accelerated metabolic disorders in mice; supplementation ameliorated them via hepatic PPARα	Human data cross-sectional; causal claims murine; unbalanced groups; single-city cohort	Indirect (constitution, not PCIV)
You et al. (33)	Chronic gastritis patients, Shanghai (disease cohort)	Disease pattern (damp-phlegm) per textbook criteria; CCMQ not used	Model 200 DP vs. 100 non-DP; external validation 50	116 differential tongue-coating metabolites; sphingolipid metabolism top-enriched; 3-metabolite classifier accuracy 94.0% (external validation 93.9%)	Single center; OPLS-DA models with negative Q^2^ reported; different disease (gastritis)	Indirect (pattern, but in gastritis, not PCIV)
Zhang et al. (29)	Systematic review/meta-analysis of 41 observational studies (11,211 participants)	Constitution; CCMQ-type scales in most included studies	41 studies	PDC pooled proportion 23% in ischemic stroke patients; over-represented vs. general population (OR 2.34, 95% CI 1.39–3.94)	Included studies low-to-moderate quality; observational designs	Indirect (stroke broadly, not PCIV; constitution level)

PDC, phlegm-dampness constitution; GC/BC, gentle/balanced constitution; CCMQ, Constitution in Chinese Medicine Questionnaire; GLU, fasting glucose; TC, total cholesterol; TG, triglycerides; LDL-C/HDL-C, low-/high-density lipoprotein cholesterol; AUC, area under the receiver-operating characteristic curve; CV, cross-validation; CAD, coronary artery disease; MAIT, mucosal-associated invariant T cell; FMT, fecal microbiota transplantation; DP, damp-phlegm pattern; OR, odds ratio.

This is a narrative, not a systematic, review. We did not perform formal risk-of-bias assessment, GRADE certainty grading, or quantitative synthesis. The review was not registered in PROSPERO, which is restricted to systematic reviews. The implications of this design, and of the indirectness of much of the evidence, are discussed where the evidence is presented as well as in Section 9.

## 3 PCIV: definition, diagnosis, and clinical context

Operational definition. In this review, PCIV denotes vertigo or dizziness attributable to ischemia in the vertebrobasilar territory. Operationally, the term as used in the literature we reviewed covers a spectrum that includes posterior circulation transient ischemic attack and infarction presenting with vertigo, and—particularly in the Chinese trial literature—patients diagnosed with “vertebrobasilar insufficiency” on the basis of symptoms and transcranial Doppler (TCD) findings without imaging-confirmed infarction. These entities are not interchangeable. The Bárány Society diagnostic criteria for vascular vertigo and dizziness provide the contemporary reference frame: they distinguish acute prolonged vascular vertigo (≥24 h) from transient vascular vertigo (minutes to <24 h), define probable and evolving categories for presentations not yet fulfilling full criteria, and cover isolated labyrinthine infarction and vertebral artery compression syndrome (17). PCIV as commonly used overlaps with, but is broader and less standardized than, these categories. The diagnostic criteria used in the clinical studies included in this review are summarized in Table 2, and the heterogeneity of those criteria is itself a limitation of the evidence base.

**Table 2:** Summary of clinical acupuncture studies in posterior circulation ischemic vertigo (PCIV).

Study (design)	Diagnostic criteria for PCIV	TCM pattern differentiation	*n*	Intervention (type; acupoints; parameters)	Comparator/co-interventions	Duration/follow-up	Main outcomes	Adverse events
Li et al. (34) (systematic review & meta-analysis of 20 RCTs)	WHO stroke criteria (1989) with imaging-confirmed posterior circulation infarction vertigo	No included trial enrolled by phlegm-dampness pattern; two trials restricted to qi/yang-deficiency patterns	1,541 (20 RCTs)	Manual acupuncture (17/20), manual + electroacupuncture (3/20); 33 main acupoints across trials, GB20 most frequent (15/20)	19/20 acupuncture + drug vs. drug alone (flunarizine, betahistine, aspirin, others); 1/20 acupuncture vs. flunarizine	7–42 days; follow-up not pooled	Basilar artery Vm SMD 0.58 (0.40–0.76); LVA 0.48 (0.22–0.73); RVA 0.44 (0.19–0.69); clinical effective rate RR 1.22 (1.15–1.29); RoB low in 6/20	7/20 trials reported safety; no acupuncture-related AEs
Dong et al. (35) (single RCT)	PCIV; detailed criteria not reported in accessible abstract	No; fixed prescription for all patients	90 (45/45)	Manual acupuncture: EX-HN3, PC6, SP6, GV20, GB20, GB12, BL10; needles retained 30 min, once daily	Flunarizine 5 mg once daily; both groups received background neurological medication	21 days; 3-month follow-up	TCM vertigo symptom score; TCD Vm and PI of LVA/RVA/BA; total effective rate 91.1% vs. 75.6%; recurrence at 3 months 19.5% vs. 50.0% (single unblinded trial)	Not reported
Wen et al. (36) (four-arm randomized safety trial)	WHO Task Force on Stroke (1989) criteria: vertigo + vertebrobasilar insufficiency on TCD/MRI ± neurological signs	Adjunct acupoints individualized by pattern (not an inclusion criterion)	136 randomized (120 completed)	Manual acupuncture at GB20 (0.5–0.8 cun), two insertion directions × two twisting frequencies (60 vs. 120/min); adjunct CV12, CV6, ST36, ST40, KI3, KI6, PC6, PC8	No drug comparator (safety design)	14 sessions over 3–4 weeks	Safety laboratory panels and ECG before/after course; adverse events after every session	5 mild events (needling pain, small hematoma, transient chest tightness); no serious AEs

Cervical/cervicogenic vertigo studies are excluded here and discussed separately as indirect evidence (Section 6.2).

Vm, mean flow velocity; PI, pulsatility index; BA, basilar artery; LVA/RVA, left/right vertebral artery; SMD, standardized mean difference; RR, relative risk; RoB, risk of bias; TCD, transcranial Doppler; AE, adverse event.

Clinical context and the place of acupuncture. Because acute or transient vascular vertigo may represent posterior circulation stroke or TIA, it is a potential medical emergency. Cerebrovascular events presenting with dizziness are disproportionately missed at initial emergency evaluation (4), and bedside oculomotor examination (HINTS) in appropriate patients is more sensitive than early MRI diffusion-weighted imaging for acute posterior circulation stroke (18). Non-contrast CT is insensitive to acute posterior fossa ischemia in the first 24 h (5). Accordingly, acupuncture should be considered only as a possible adjunct after appropriate diagnosis and stabilization; it must not delay stroke evaluation, acute reperfusion therapy where indicated, or secondary prevention. Safety considerations specific to this population include bleeding risk in patients taking antiplatelet or anticoagulant therapy, and the anatomical hazards of needling the upper cervical region: acupoints frequently used for PCIV, such as GB20, GB12, and BL10, lie close to the vertebral artery and the medulla, and case reports document serious injuries from deep or misdirected suboccipital needling (19, 20). Needling in this region should follow standardized depth and direction guidance and be performed by trained practitioners.

Epidemiology. Posterior circulation events account for approximately 20–25% of ischemic strokes (1–3), and registries and classic reviews confirm that vertigo and dizziness are among their commonest presentations (3, 21, 22). The prevalence of a vertigo-dominant PCIV syndrome as a distinct clinical entity, however, is not well established in population-based data, and claims about its frequency, recurrence, and misdiagnosis rates should be read against the diagnostic heterogeneity described above.

## 4 Phlegm-dampness in PCIV: traditional theory and clinical pattern evidence

### 4.1 Classical conceptualization—as historical context

Within TCM theory, vertigo arises from an interplay among wind, fire, phlegm, and deficiency, with phlegm receiving particular classical emphasis (6–8). In the classical account, impaired splenic transformation of fluids leads to dampness accumulation, which condenses into phlegm, obstructs the middle jiao, and prevents clear yang from ascending to nourish the head; the described clinical correlate is rotational vertigo aggravated by movement, heavy-headedness, chest oppression, nausea, fatigue, a pale enlarged tongue with greasy coating, and a slippery pulse (8, 23). We present this framework as the historical and theoretical rationale for studying phlegm-dampness in PCIV. Classical texts cannot serve as evidence for the frequency, biology, or treatment response of any pattern in a modern clinical population, and we do not use them as such below.

### 4.2 Constitution is not pattern: an essential distinction

Two related but distinct TCM constructs appear in the modern literature, and conflating them has been a source of confusion in interpreting biomarker findings. Constitution (体质) refers to a relatively stable, trait-like predisposition assessed in health or disease; the 2009 China Association of Chinese Medicine standard (ZYYXH/T157-2009) and the Constitution in Chinese Medicine Questionnaire (CCMQ) operationalize nine constitution types, including phlegm-dampness constitution (7, 24). A disease pattern (证候, syndrome), by contrast, is a context-dependent clinical classification assigned during an episode of illness; it can change as the illness evolves or responds to treatment, and pattern differentiation for PCIV in practice follows textbook and guideline conventions rather than a single internationally standardized instrument (6, 25). The two constructs may be related—a phlegm-dampness constitution may predispose to phlegm-dampness patterns during illness—but they are not interchangeable, and the 2009 constitution criteria must not be read as standardized diagnostic criteria for phlegm-dampness pattern in PCIV. This distinction matters because nearly all biomarker evidence reviewed in Section 5 was obtained in constitution-defined cohorts, not in pattern-defined PCIV patients.

### 4.3 What clinical evidence exists on pattern distribution in PCIV?

Direct evidence on the distribution of TCM disease patterns in PCIV is limited and of low methodological quality. The largest dedicated survey, a Chinese master’s thesis studying 350 PCIV patients with cluster and factor analysis, reported phlegm-turbidity obstruction (痰浊中阻) as the most frequent pattern (22.0%), followed by qi-blood deficiency (18.3%), liver-yang hyperactivity (14.9%), and phlegm-stasis obstruction (14.0%) (26). Two smaller angiography- or imaging-anchored studies in posterior circulation ischemic stroke provide convergent observations: among 55 patients with DSA-confirmed posterior circulation ischemic stroke, wind-phlegm obstruction of the collaterals (风痰阻络) was the most frequent pattern (22.0%) (27), and in an Oxfordshire Community Stroke Project-classified cohort of 225 acute stroke patients, wind-phlegm obstruction accounted for 54.7% of the posterior circulation infarction subgroup (47 of 86) (28). At the constitution level, a systematic review and meta-analysis of 41 observational studies (11,211 participants) found phlegm-dampness constitution among the commonest constitution types in ischemic stroke patients (pooled proportion 23, 95% CI 0.20–0.29) and significantly over-represented relative to the general population (pooled odds ratio 2.34, 95% CI 1.39–3.94), although the included studies were of low to moderate quality (29).

These data suggest that phlegm-related patterns are commonly assigned in posterior circulation ischemic conditions, but they do not establish that phlegm-dampness is the most frequent pattern in objectively diagnosed PCIV: the studies are small, single-country, use heterogeneous diagnostic and pattern-differentiation criteria, and include thesis-level evidence. We therefore present the prominence of phlegm-dampness in PCIV as a traditional theoretical proposition with preliminary clinical support, not as established epidemiology, and we have removed statements to the contrary from the Abstract and Introduction.

## 5 The biological substrate of phlegm-dampness constitution: evidence from non-PCIV cohorts

This section reviews metabolic, immunological, transcriptomic, and microbiome findings associated with phlegm-dampness. Critically, almost all of this evidence derives from phlegm-dampness constitution cohorts—generally community or clinic volunteers without PCIV—or from disease cohorts other than PCIV. Whether PCIV patients assigned a phlegm-dampness disease pattern share any of these abnormalities is unknown, and none of the findings below should be read as direct evidence about PCIV. Table 1 summarizes each study’s population, instrument, sample size, key findings, limitations, and directness to PCIV.

### 5.1 Metabolic phenotype

Phlegm-dampness constitution is associated with a metabolic profile that overlaps with metabolic syndrome. In an integrated diagnostic-model study of 74 phlegm-dampness constitution and 66 balanced-constitution subjects, the phlegm-dampness group displayed higher fasting glucose, total cholesterol, triglycerides, and low-density lipoprotein cholesterol with reduced high-density lipoprotein cholesterol; a stepwise logistic-regression model combining fasting glucose, triglycerides, low-density lipoprotein cholesterol, and lymphocyte count discriminated phlegm-dampness from balanced constitution with an area under the receiver-operating characteristic curve of 0.878 (11). This figure requires careful interpretation. It was derived in a constitution-defined sample of cardiology outpatients and health-screening attendees, not in PCIV patients; the model was fitted and evaluated on the same 140 subjects, with no training–validation split, cross-validation, or external validation, so 0.878 is an optimistic in-sample estimate; and the phlegm-dampness group carried substantially more diabetes (41.9% vs. 10.6%) and coronary artery disease (15.6% vs. 1.5%) than controls, a confounder that the model does not address. An in-sample AUC of this kind quantifies discrimination between two constitution groups within one cohort; it does not establish a biological mechanism, a disease-specific endotype, or applicability to PCIV. Independent proteomic work reports concordant directional changes in lipid-metabolism and insulin-resistance pathways in phlegm-dampness constitution, including subclinical dyslipidemia in subjects whose routine blood tests were within reference ranges (30).

### 5.2 Immunological and transcriptomic associations

In a single-cell RNA-sequencing study of peripheral blood mononuclear cells from phlegm-dampness constitution subjects, Zhao and colleagues observed reduced mucosal-associated invariant T-cell content together with heightened TNF-α–NF-κB, JAK–STAT, and interferon signaling, accompanied by increased hypoxia and apoptosis responses (10). This is a single cross-sectional study of modest size; the MAIT-cell finding in particular should be regarded as preliminary and hypothesis-generating rather than an established feature of phlegm-dampness constitution. Long non-coding RNA and mRNA profiling of peripheral blood mononuclear cells has likewise identified differentially expressed transcripts enriched in lipid-metabolism and immune–inflammation pathways, suggesting associated transcriptomic profiles—a cross-sectional association that cannot establish stable or causal programs (31). ELISA-based studies are consistent with a low-grade inflammatory phenotype, although effect sizes vary across cohorts (11). Multi-omics reviews of TCM syndromes in stroke reach a similar conclusion at the field level: recurring metabolic and inflammatory signals, but heterogeneous methods and little external replication (32).

### 5.3 Gut microbiome

The most mechanistically developed evidence linking phlegm-dampness constitution to a defined biological pathway comes from gut-microbiome work. Li and colleagues showed that subjects with phlegm-dampness constitution harbor distinct fecal microbial communities and serum metabolic profiles, with a marked reduction in *Flavonifractor plautii* and its metabolic product phytosphingosine (9). Phytosphingosine levels correlated negatively with phlegm-dampness scores and metabolic-disorder severity. Fecal microbiota transplantation from phlegm-dampness subjects accelerated metabolic disorders in recipient mice, and supplementation with *F. plautii* or phytosphingosine ameliorated these disorders by activating hepatic PPARα (9). The human component of this work is associational and was performed in constitution cohorts; the causal component is murine. Tongue-coating metabolomic studies provide an additional, anatomically distinct line of evidence for differential lipid and sphingolipid metabolism in phlegm-dampness states—in that case assessed as a disease pattern in chronic gastritis, again outside PCIV (33).

### 5.4 Interim summary

Considered together, these findings describe a convergent but not yet independently replicated—and still constitution-level—metabolic–microbial signature of phlegm-dampness: dyslipidemia, microbiome-driven metabolic dysregulation, and low-grade systemic inflammation. Two inferential steps remain unproven: first, that PCIV patients with a phlegm-dampness disease pattern exhibit this signature; and second, that the signature defines a subgroup with distinct treatment response. The framework in Section 8 is therefore offered as a candidate endotype model that makes these steps explicit and testable, not as an established endotype.

## 6 Clinical evidence for acupuncture in PCIV

This section separates direct evidence obtained in PCIV populations (Section 6.1) from indirect evidence obtained in cervical or cervicogenic vertigo (Section 6.2), and then appraises the methodological limits of both (Section 6.3). Table 2 summarizes the clinical PCIV studies, including their diagnostic criteria, interventions, and outcomes.

### 6.1 Direct evidence in PCIV

The most comprehensive PCIV-specific synthesis to date is the 2022 meta-analysis by Li and colleagues, which pooled 20 randomized controlled trials with 1,541 participants and reported significant improvements in basilar artery (standardized mean difference [SMD] = 0.58, 95% CI 0.40–0.76), left vertebral artery (SMD = 0.48, 95% CI 0.22–0.73), and right vertebral artery (SMD = 0.44, 95% CI 0.19–0.69) blood-flow velocities, together with a higher clinical effective rate (relative risk [RR] = 1.22, 95% CI 1.15–1.29) for acupuncture—usually as an adjunct to conventional pharmacotherapy—compared with conventional therapy alone (34). The included trials diagnosed posterior circulation infarction vertigo by WHO stroke criteria with imaging confirmation; treatments were predominantly manual acupuncture (17 of 20 trials) over 7–42 days, with GB20 the most frequently used acupoint (15 of 20 trials). Risk of bias was low in only 6 of 20 trials, no trial was participant- or assessor-blinded, and no included trial enrolled patients by phlegm-dampness—or any single phlegm-related—pattern. The journal in which this meta-analysis appeared has since been removed from the Web of Science Core Collection; independent of indexing status, the underlying trials’ risk-of-bias profile alone requires that the pooled estimates be read with caution.

At the level of individual protocols, the “Xing Nao Kai Qiao” protocol—combining Yintang (EX-HN3), Neiguan (PC6), Sanyinjiao (SP6), Baihui (GV20), Fengchi (GB20), Wangu (GB12), and Tianzhu (BL10), delivered as manual acupuncture once daily for 21 days—improved TCM vertigo-symptom scores and TCD-measured posterior-circulation flow velocities compared with flunarizine in a single 90-patient randomized trial (35). That trial also reported lower recurrence at 3-month follow-up in the acupuncture group (19.5% vs. 50.0%), but this is a single, small, unblinded study, and no pooled recurrence estimate exists; we have therefore removed recurrence from the outcomes presented in Figure 1 and treat durability of effect as an open question. A four-arm randomized safety study in 136 PCIV patients found that needling GB20 at either of two insertion directions and two twisting frequencies over 14 sessions was associated with only mild, transient adverse events when performed by qualified practitioners (36).

Interpreting the hemodynamic outcomes requires care. TCD measures blood-flow velocity, not volumetric cerebral blood flow, tissue perfusion, cerebral oxygen delivery, or restored autoregulation; velocity is influenced by vessel diameter, stenosis, carbon dioxide tension, systemic blood pressure, and measurement technique (37, 38). The pooled velocity increases are therefore best interpreted as evidence of a hemodynamic signal, not as demonstrated restoration of posterior-circulation perfusion or autoregulatory function. The “clinical effective rate” pooled in these meta-analyses is likewise problematic: it is a non-standardized composite of subjective symptom categories defined per Chinese trial conventions, varies across trials, and is particularly vulnerable to bias in unblinded studies using subjective criteria (34). The clinical evidence thus supports a probable symptomatic and hemodynamic benefit of low certainty; it does not yet establish benefit on patient-important outcomes such as stroke recurrence, falls, or vestibular function measured objectively.

### 6.2 Indirect evidence from cervical and cervicogenic vertigo

A larger body of randomized evidence concerns cervical or cervicogenic vertigo. This population is not synonymous with PCIV: patients labelled as having cervical vertigo may have cervical proprioceptive dysfunction, musculoskeletal symptoms, vestibular disorders, uncertain mechanisms, or vascular disease, and the assumption of shared vertebrobasilar pathophysiology is not generally justified. We therefore report these studies separately and treat them strictly as indirect evidence. A 2025 PRISMA-2020-compliant systematic review and meta-analysis by Yang and colleagues evaluated acupuncture combined with Western medicine in cervical vertigo (39). Across seven RCTs (714 participants), combination therapy outperformed Western medicine alone in left vertebral, right vertebral, and basilar artery flow velocities, in cervical-vertigo symptom and function scores, and in clinical efficacy; trial sequential analysis indicated that the cumulative evidence had crossed pre-specified information thresholds for the primary outcomes (39). A 2026 network meta-analysis of 66 RCTs (5,797 patients) found that combinations of electroacupuncture with Tuina or moxibustion outperformed monotherapy for cervicogenic vertigo, although the certainty of evidence remained low to very low (40). Earlier meta-analyses similarly favor acupuncture for cervical vertigo, with the same caveat of low to very low certainty on GRADE assessment (41). A 2026 meta-analysis of acupuncture combined with bone-setting (Tuina) in 15 RCTs (n = 2,320) reported higher overall clinical efficacy than either modality alone (odds ratio 3.88, 95% CI 2.89–5.19) (42). Because the intervention in these combination studies is a package of acupuncture with Tuina, bone-setting, or moxibustion, their results cannot be attributed to acupuncture alone; and because the population is not PCIV, none of these findings should be generalized to PCIV without direct confirmation.

### 6.3 Methodological limits of the current evidence base

Three methodological problems recur across the PCIV acupuncture literature and constrain inference. First, most trials originate from a single national context, and independent replication is rare. Second, sham-acupuncture or active comparator controls are uncommon, leaving open the contribution of non-specific therapeutic effects; in other pain conditions, adequately sham-controlled individual-patient-data meta-analyses show that a substantial share of acupuncture’s effect is attributable to such non-specific factors, and no equivalent analysis exists for PCIV (43). Third, vestibular-specific outcomes—video head impulse test (vHIT), cervical and ocular vestibular evoked myogenic potentials, and caloric testing—are seldom used; clinical efficacy continues to depend on composite symptom scales whose response to acupuncture is large but plausibly inflated by non-blinded outcome assessment. These limits do not invalidate current conclusions, but they define the agenda for next-generation trials.

## 7 Mechanistic pathways: what acupuncture could plausibly modify

This section reviews four mechanistic axes through which acupuncture could plausibly act in PCIV, integrating the acupoint-level discussion with the pathway-level discussion to avoid duplication. Two qualifications apply throughout. First, for each axis we state the type of evidence available: direct human evidence in pattern-defined PCIV does not exist for any of these pathways; the strongest human evidence concerns hemodynamics in PCIV patients without pattern classification and in healthy volunteers, while inflammatory, antioxidant, and gut–brain mechanisms are supported predominantly by rodent studies. Second, showing that acupuncture changes a biomarker in an animal model is not equivalent to demonstrating that the marker mediates clinical improvement in patients. Acupoint nomenclature and locations in this review follow the WHO standard (44).

### 7.1 Cerebrovascular hemodynamics of the posterior circulation

Evidence base: human studies in PCIV patients (without pattern classification) and healthy volunteers; animal endothelial studies.

In healthy volunteers, TCD studies have shown that acupuncture at head and neck acupoints modifies cerebrovascular CO₂ reactivity in the basilar and middle cerebral arteries without altering systemic blood pressure or heart rate, consistent with a local vascular rather than a systemic vasomotor effect (37, 38, 45). In PCIV patients, acupuncture protocols centered on GB20 with adjunct neck and scalp points are associated with improved vertebrobasilar flow velocities on TCD, as summarized in Section 6.1 (34, 35). Candidate mediators include endothelial nitric oxide signaling—electroacupuncture improved cerebral blood flow and attenuated ischemic injury through an endothelium-dependent mechanism in mice—and modulation of perivascular sympathetic tone (46, 47). These findings should be read with the measurement caveats of Section 6.1: TCD velocity and CO₂ reactivity are intermediate physiological measures, not tissue perfusion, and no study has linked acupuncture-induced velocity changes to restored autoregulation or to clinical outcomes in PCIV.

Acupoint specificity—GB20 (Fengchi). GB20 is the most frequently used acupoint in PCIV trials (15 of 20 trials in the PCIV meta-analysis) and lies in the suboccipital depression superficial to the V3 segment of the vertebral artery (34). In healthy volunteers, GB20 acupuncture modified basilar-artery CO₂ reactivity more than middle-cerebral-artery reactivity (37). Anatomical proximity to the vertebral artery, however, does not by itself establish direct or selective modulation of posterior-circulation hemodynamics: central sensory, autonomic, systemic, and measurement-related mechanisms remain plausible alternatives, and the available volunteer studies cannot exclude them. The suboccipital location also carries safety implications: needling depth, direction, and anatomical variation in this region demand standardized technique, and serious injuries from deep suboccipital needling have been reported in the safety literature (Section 3) (19, 20, 36).

### 7.2 Suppression of NF-κB- and NLRP3-driven neuroinflammation

Evidence base: rodent cerebral ischemia models (anterior circulation); no human PCIV data.

NF-κB is a master regulator of inflammatory gene expression in the ischemic brain (48). In rat middle cerebral artery occlusion (MCAO) models, electroacupuncture at GV20, LI4, and LR3 upregulates the deubiquitinase A20 and the A20-binding inhibitor of NF-κB activation 1 (ABIN1) in peri-infarct cortex, attenuating NF-κB activation, microglial M1 polarization, and pro-inflammatory cytokine production; loss-of-function manipulations of ABIN1 partially abolish these benefits, supporting a causal role within the model (49, 50). Related work reports NF-κB-mediated microglial suppression as a shared mechanism across electroacupuncture studies in ischemic stroke models (51, 52). The NLRP3 inflammasome is a parallel hub linking metabolic stress to sterile neuroinflammation (53). Electroacupuncture inhibits NLRP3 inflammasome activation in rodent ischemia–reperfusion models, reducing NLRP3, ASC, cleaved caspase-1, mature IL-1β, and gasdermin-D-mediated pyroptosis (15, 54, 55), and this inhibition can be mediated through α7 nicotinic acetylcholine receptors (α7nAChR), consistent with the cholinergic anti-inflammatory pathway (56). Two cautions apply. First, these experiments were conducted in anterior-circulation (MCAO) or ischemia–reperfusion models, not in models of posterior-circulation ischemia or vertebrobasilar insufficiency; NLRP3 suppression in an MCAO rat does not establish NLRP3 as the mechanism of benefit in vascular vertigo. Second, the relevance to phlegm-dampness PCIV is inferential: oxidized lipids, cholesterol crystals, and hyperglycemia can prime and activate NLRP3 (53), which provides biological plausibility for a metabolic–inflammatory link, but that link has not been tested in pattern-defined patients.

### 7.3 Antioxidant defense via the Nrf2/ARE pathway

Evidence base: rodent models of vascular dementia and MCAO; no human PCIV data.

Nrf2 is the principal transcriptional regulator of the cellular antioxidant response (57). In a foundational study, Wang and colleagues showed that the cognitive and neuronal-protective effects of acupuncture in experimental vascular dementia were abolished in Nrf2-knockout mice, with wild-type mice showing acupuncture-induced upregulation of HO-1 and NQO1 in hippocampal tissues (14). Subsequent work extended Nrf2-dependent mechanisms to electroacupuncture-mediated suppression of ferroptosis through the Nrf2/SLC7A11/GPX4 axis after MCAO (58). Systematic reviews of preclinical antioxidant outcomes for acupuncture in cerebrovascular models report consistent reductions in reactive oxygen species and malondialdehyde, with corresponding increases in superoxide dismutase and glutathione peroxidase, although study quality is heterogeneous (59). Electroacupuncture at GV20 (with GV14)—the vertex acupoint localized in imaging work beneath the cerebral cortex (60)—also elevates BDNF, VEGF, and SDF-1α expression and reduces infarct volume in rodent ischemia models, overlapping with the antioxidant and neurotrophic programs above (61, 62). Whether oxidative-stress markers are relevant to phlegm-dampness PCIV specifically—beyond generic ischemia biology—has not been examined in any population.

### 7.4 Gut–brain axis and systemic metabolic regulation

Evidence base: rodent stroke and hyperlipidemia models, plus one mechanistic study in mice with defined stimulation conditions; no human PCIV data.

Liu and colleagues demonstrated that low-intensity electroacupuncture at hindlimb acupoints (notably ST36) recruits PROKR2-Cre-marked deep-fascia sensory neurons to drive a vagal–adrenal anti-inflammatory axis, providing a defined neuroanatomical substrate for somatosensory–autonomic regulation by acupuncture (12). This pathway was demonstrated under specific experimental conditions—low-intensity electroacupuncture in mice—and is body-region- and intensity-dependent; it should not be generalized to all forms of manual acupuncture or to clinical stimulation at ST36. The gut microbiome provides a complementary mechanism. Electroacupuncture at LI11 and ST36 altered gut microbial composition and increased acetate, propionate, and total short-chain fatty acid (SCFA) levels in peripheral blood after MCAO in rats, with SCFA changes correlating with motor recovery and infarct size (63). Reviews of acupuncture–microbiome interactions in stroke describe additional candidate pathways, including modulation of trimethylamine-N-oxide, indole-3-propionic acid, and Th17/Treg balance (13, 64–66). Increased SCFAs in an MCAO rat, however, do not demonstrate restoration of the *F. plautii*–phytosphingosine pathway in PCIV patients; that specific connection is a hypothesis, not an observation.

Acupoint specificity—ST36 (Zusanli) and ST40 (Fenglong). ST36 is the most extensively characterized acupoint in modern neurobiological studies of acupuncture (12) and modulated cerebrovascular reactivity in one healthy-volunteer study (45). ST40 is the traditional acupoint for phlegm transformation and the Luo-connecting point of the stomach meridian (23). In hyperlipidemic rats and ApoE-knockout mice, electroacupuncture at ST40 reduced total cholesterol and LDL-cholesterol, modulated ABCA1, PPARα, and LXRα expression in liver and macrophages, attenuated foam-cell formation, and inhibited hepatic IL-17 expression (67, 68). These lipid-regulatory data are entirely preclinical: no human study has shown that ST40 stimulation improves dyslipidemia, and none has examined it in PCIV. The inclusion of ST36 or ST40 in prescriptions for phlegm-dampness PCIV therefore currently rests on traditional indication plus animal evidence, not on human mechanistic or outcome data.

## 8 A candidate metabolic–microbial endotype framework

Synthesizing Sections 3–7, we propose a candidate endotype framework as an explicit, falsifiable hypothesis (Figure 1 and Table 3). The proposal is that PCIV patients assigned a phlegm-dampness pattern may constitute a subgroup characterized at four levels: (i) a metabolic phenotype of dyslipidemia and insulin resistance; (ii) a microbial signature featuring depletion of *Flavonifractor plautii* and its product phytosphingosine; (iii) a low-grade inflammatory–oxidative state; and (iv) a vascular manifestation of impaired posterior-circulation hemodynamics.

**Table 3:** The candidate metabolic–microbial endotype framework, with the source population, evidence type, and directness to PCIV stated for each proposed relationship.

Proposed level	Phenotypic features (source population)	Evidence type and directness to PCIV	Proposed acupuncture axis (evidence type)	Representative acupoints (evidence base)	Key limitations
(i) Metabolic	↑ TC, LDL-C, TG; ↓ HDL-C; ↑ fasting glucose; ↑ HOMA-IR (phlegm-dampness constitution cohorts) (11, 30)	Human, constitution-defined, cross-sectional; indirect to PCIV	Lipid regulation via ABCA1, PPARα, LXRα; reduced foam-cell formation; hepatic IL-17 modulation (preclinical: hyperlipidemic rats, ApoE-knockout mice) (67, 68)	ST40, PC6 (animal evidence only; ST40 not reported in PCIV trials)	Features not confirmed in pattern-defined PCIV; no human evidence that acupuncture improves dyslipidemia in PCIV
(ii) Microbial	↓ *Flavonifractor plautii*; ↓ phytosphingosine (constitution cohorts; causal data murine) (9)	Human association + mouse FMT; indirect to PCIV	↑ Acetate, propionate, total SCFAs; microbial composition modulation (preclinical MCAO rats) (63); PROKR2-Cre vagal–adrenal axis (low-intensity electroacupuncture, mice) (12)	ST36 + LI11 (animal evidence only)	*F. plautii*–phytosphingosine pathway never studied after acupuncture or in PCIV; vagal–adrenal findings not generalizable to manual acupuncture
(iii) Inflammatory–oxidative	↑ TNF-α–NF-κB, JAK–STAT, interferon signaling; ↓ MAIT cells (single scRNA-seq study); associated transcriptomic profiles (constitution cohorts) (10, 31)	Human, constitution-defined, cross-sectional; indirect to PCIV	↓ NF-κB via A20/ABIN1; ↓ NLRP3 inflammasome (α7nAChR-mediated); ↓ pyroptosis (preclinical MCAO/ischemia–reperfusion) (49, 50, 54, 56); ↑ Nrf2/HO-1/NQO1, ↓ ferroptosis (preclinical vascular dementia/MCAO) (14, 58)	GV20 + LI4 + LR3; GV20 + GV14 (animal evidence only)	Anterior-circulation models; no PCIV or pattern-stratified data; MAIT finding unreplicated; oxidative phenotype not measured in phlegm-dampness cohorts
(iv) Vascular	↓ Vertebrobasilar flow velocity on TCD (PCIV patients without pattern classification) (34)	Human PCIV evidence; direct to the condition, not to the pattern	↑ BA/LVA/RVA flow velocity (human PCIV trials; healthy-volunteer TCD studies) (34, 36, 37); endothelial NO signaling (mice) (46)	GB20 ± GB12, BL10 within multi-point protocols (used in PCIV trials) (34, 35)	TCD velocity ≠ volumetric flow, perfusion, or autoregulation; the GB20 + GB12 + BL10 combination not independently evaluated; no link from velocity change to clinical outcome

All mappings are hypotheses; none has been validated in pattern-defined PCIV patients.

TC, total cholesterol; LDL-C, low-density lipoprotein cholesterol; TG, triglycerides; HDL-C, high-density lipoprotein cholesterol; HOMA-IR, homeostatic model assessment of insulin resistance; SCFA, short-chain fatty acid; PPARα, peroxisome proliferator-activated receptor α; LXRα, liver X receptor α; ABCA1, ATP-binding cassette transporter A1; MAIT, mucosal-associated invariant T cell; NF-κB, nuclear factor kappa B; ABIN1, A20-binding inhibitor of NF-κB activation 1; NLRP3, NOD-like receptor protein 3; α7nAChR, α7 nicotinic acetylcholine receptor; HO-1, heme oxygenase-1; NQO1, NAD(P)H quinone oxidoreductase 1; GPX4, glutathione peroxidase 4; SLC7A11, solute carrier family 7 member 11; TCD, transcranial Doppler; BA, basilar artery; LVA/RVA, left/right vertebral artery; MCAO, middle cerebral artery occlusion; FMT, fecal microbiota transplantation; NO, nitric oxide.

Three qualifications define the current status of this framework. First, the components come from separate studies in different populations—constitution cohorts, PCIV patients without pattern classification, healthy volunteers, and animal models—and have never been measured jointly in pattern-defined PCIV patients; whether they co-occur, distinguish this subgroup from other-pattern PCIV patients, or remain associated after adjustment for metabolic confounders is unknown. Second, the correspondence between the four levels and acupuncture’s mechanistic axes is a proposed mapping, not a demonstrated one: the hemodynamic axis rests on human (albeit pattern-unclassified) evidence, whereas the inflammatory, antioxidant, and gut–brain axes rest on preclinical evidence. Third, no trial has tested whether phlegm-dampness-pattern PCIV patients respond differently to acupuncture; our targeted search found no randomized acupuncture trial in PCIV that enrolled patients by phlegm-dampness pattern (Section 6.1).

The value of the framework is that it generates testable predictions. First, it predicts that phlegm-dampness PCIV patients should respond more favorably to acupuncture than other-pattern PCIV patients matched for clinical severity—a hypothesis that, to our knowledge, has not been directly tested in adequately powered head-to-head trials. Second, it identifies a candidate biomarker panel (lipid profile, HOMA-IR, an inflammatory composite, and a microbiome-based signature centered on phytosphingosine-producing taxa) for stratification in future trials—to be validated first in pattern-defined PCIV cohorts before use as a stratification tool. Third, it suggests that the durability of acupuncture’s effect should track the durability of biomarker change, providing an objective endpoint less vulnerable to non-blinding bias than symptom scales. If validated, pattern-based prescribing in this subgroup could come to resemble endotype-informed therapy; that prospect is currently a research agenda, not a clinical recommendation.

## 9 Limitations

Several limitations constrain the conclusions of this review. First, this is a narrative rather than a systematic review; selection of studies, although guided by SANRA principles and an explicit search strategy, was not subjected to formal risk-of-bias scoring or quantitative synthesis. Selection bias toward positive mechanistic findings cannot be excluded. Second, much of the clinical evidence for acupuncture in PCIV originates from a single national context, often without sham control or independent replication. Some included work appeared in journals subsequently delisted from major indexing services; we have treated indexing status not as a quality criterion in itself but as a prompt to appraise each study’s design and reporting individually. Third, the proposed framework is hypothesis-generating: although each of its components is supported by independent evidence, the evidence tiers differ—human constitution cohorts for the biomarker levels, human PCIV trials without pattern classification for the hemodynamic level, and animal models for most mechanistic links—and the predicted differential response to acupuncture across pattern subtypes has not been directly tested. Fourth, vestibular-specific functional outcomes are sparsely reported in the existing literature, limiting our ability to assess effects on the structures most directly responsible for vertigo. Fifth, mechanistic evidence remains heavily preclinical; translation to human responders will require deliberate biomarker integration in subsequent trials.

Finally, the reproducibility and external applicability of the proposed framework are uncertain. The biomarker findings derive almost entirely from Chinese phlegm-dampness constitution cohorts, whose diet, microbiome background, and metabolic risk profiles may differ from other populations; TCM pattern differentiation itself has imperfect inter-rater reliability and is applied differently across schools and settings; and the acupuncture protocols reviewed here were developed and evaluated within a single healthcare system. Until the candidate endotype is tested in independent, multi-center, and ideally multi-ethnic cohorts using standardized pattern-diagnostic procedures, the framework should be regarded as transportable only as a hypothesis, not as a validated stratification tool.

## 10 Future directions

Three priorities follow from the framework above. First, pattern-stratified pragmatic trials should test whether phlegm-dampness PCIV patients show larger or more durable benefit from acupuncture than other-pattern PCIV patients, using pre-specified biomarker panels for stratification. Second, vestibular-specific outcomes—vHIT, cervical and ocular vestibular evoked myogenic potentials, and standardized dizziness handicap measures—should be incorporated alongside hemodynamic endpoints, and TCD velocity should be supplemented or replaced by measures closer to tissue perfusion where feasible. Third, mechanistic substudies nested within clinical trials should track lipid profiles, HOMA-IR, inflammatory cytokines, and microbial composition longitudinally, enabling causal-inference work on whether acupuncture’s clinical benefit is mediated by endotype-specific biomarker change. Standardization of electroacupuncture parameters in line with the STandards for Reporting Interventions in Clinical Trials of Acupuncture (STRICTA), together with adequately sham-controlled designs, remains essential (69).

## 11 Conclusion

Current evidence permits three conclusions. First, phlegm-dampness occupies a central place in traditional vertigo theory and appears commonly assigned in posterior circulation ischemic conditions, but robust PCIV-specific pattern-frequency data are lacking. Second, phlegm-dampness constitution—assessed outside PCIV—carries a convergent but not yet independently replicated metabolic, inflammatory, and microbial signature, while the clinical evidence for acupuncture in PCIV shows low-certainty hemodynamic and symptomatic benefit. Third, integrating these strands into a candidate metabolic–microbial endotype framework yields explicit, testable predictions for biomarker-stratified trials. Whether phlegm-dampness pattern in PCIV truly denotes a biological endotype, and whether acupuncture offers endotype-specific benefit, are questions that only pattern-stratified, biomarker-embedded clinical studies can answer.
