# Supplementary material for: Toward precision acupuncture: phlegm-dampness pattern as a candidate metabolic–microbial endotype in posterior circulation ischemic vertigo: a narrative review
Source: Front Neurol. 2026 Aug 24;17:1876704. doi: 10.3389/fneur.2026.1876704 (PMC13550721; doi:10.3389/fneur.2026.1876704)
Supplement: Supplementary file 1 [file Table_1.DOCX]

**Supplementary Table S1. Representative complete search strategy (PubMed).**

Search period: inception to 31 October 2025; update search of all six databases completed 30 June 2026. No language restrictions were applied. The same concept blocks were adapted for Embase, Web of Science, the Cochrane Library, CNKI, and Wanfang (English and Chinese terms as appropriate).

| **Block 1 — acupuncture** | ("acupuncture"[MeSH Terms] OR "acupuncture therapy"[MeSH Terms] OR acupuncture[tiab] OR electroacupuncture[tiab] OR "manual acupuncture"[tiab] OR acupoint*[tiab] OR moxibustion[tiab]) |
| --- | --- |
| **Block 2 — condition** | ("posterior circulation"[tiab] OR vertebrobasilar[tiab] OR "basilar artery"[tiab] OR "vertebral artery"[tiab] OR "posterior circulation infarct*"[tiab] OR "ischemic vertigo"[tiab] OR "vascular vertigo"[tiab] OR "cervical vertigo"[tiab] OR "cervicogenic dizziness"[tiab] OR "vertigo"[MeSH Terms] OR "dizziness"[MeSH Terms]) |
| **Block 3 — mechanism/pattern** | ("phlegm-dampness"[tiab] OR "phlegm dampness"[tiab] OR Tanshi[tiab] OR "gastrointestinal microbiome"[MeSH Terms] OR microbiome[tiab] OR "short-chain fatty acid*"[tiab] OR SCFA[tiab] OR "NF-kappa B"[MeSH Terms] OR NLRP3[tiab] OR Nrf2[tiab] OR "cerebral blood flow"[tiab] OR "cerebrovascular circulation"[MeSH Terms] OR vagal[tiab] OR "vagus nerve"[MeSH Terms] OR endotype*[tiab] OR "metabolic syndrome"[MeSH Terms]) |
| **Combined** | Block 1 AND Block 2 AND Block 3. Chinese databases searched with the corresponding Chinese terms: 针灸/电针/针刺/穴位; 后循环缺血/椎基底动脉供血不足/眩晕/颈性眩晕; 痰湿/体质/证型/肠道菌群/短链脂肪酸. |

Note: The constitution-based biomarker studies (Section 5 of the manuscript) and the non-PCIV mechanistic studies (Section 7) were additionally identified through supplementary targeted searches of the same databases using construct- and pathway-specific terms, and through screening of the reference lists of included articles and relevant reviews. The strategy above is therefore the representative core strategy for the PCIV-focused search, not an exhaustive record of every supplementary search (see Section 2 of the manuscript).
